# Understanding the factors influencing health-worker employment decisions in South Africa

**DOI:** 10.1186/1478-4491-11-15

**Published:** 2013-04-23

**Authors:** Gavin George, Jeff Gow, Shaneel Bachoo

**Affiliations:** 1 Health Economics and HIV and AIDS Research Division, University of KwaZulu-Natal, Private Bag X54001, Durban, 4000, South Africa; 2 School of Accounting, Economics and Finance, University of Southern Queensland, Toowoomba, QLD, 4350, Australia

**Keywords:** Health workers, Human resources for health, Public sector, Private sector, Nongovernmental organization sector, KwaZulu-Natal, South Africa

## Abstract

**Background:**

The provision of health care in South Africa has been compromised by the loss of trained health workers (HWs) over the past 20 years. The public-sector workforce is overburdened. There is a large disparity in service levels and workloads between the private and public sectors. There is little knowledge about the nonfinancial factors that influence HWs choice of employer (public, private or nongovernmental organization) or their choice of work location (urban, rural or overseas). This area is under-researched and this paper aims to fill these gaps in the literature.

**Method:**

The study utilized cross-sectional survey data gathered in 2009 in the province of KwaZulu-Natal. The HWs sample came from three public hospitals (*n* = 430), two private hospitals (*n* = 131) and one nongovernmental organization (NGO) hospital (*n* = 133) in urban areas, and consisted of professional nurses, staff nurses and nursing assistants.

**Results:**

HWs in the public sector reported the poorest working conditions, as indicated by participants’ self-reports on stress, workloads, levels of remuneration, standard of work premises, level of human resources and frequency of in-service training. Interesting, however, HWs in the NGO sector expressed a greater desire than those in the public and private sectors to leave their current employer.

**Conclusions:**

To minimize attrition from the overburdened public-sector workforce and the negative effects of the overall shortage of HWs, innovative efforts are required to address the causes of HWs dissatisfaction and to further identify the nonfinancial factors that influence work choices of HWs. The results highlight the importance of considering a broad range of nonfinancial incentives that encourage HWs to remain in the already overburdened public sector.

## Background

In the health-care field, addressing the health care needs of a population is largely dependent on the provision of effective, efficient and high-quality health services, and the health workforce provides arguably the most important contribution to this process [1]. In 2008, there were approximately 250 000 health workers (HWs) employed in South Africa’s health sector; relatively the same amount of HWs as in 1997/98 [2]. When taking into account population growth and the burden of disease, the Development Bank of South Africa calculated a staff shortage of 80 000 HWs [3]. This critical shortage of HWs is being experienced at a time when the population is increasing and the burden of ill-health, primarily due to HIV, AIDS and tuberculosis, is also on the rise [2]. The South African health system comprises a strong private sector, serving less than one-fifth of the population but employing 70% of medical doctors and 54% of professional nurses [4-6]. Furthermore, there are large disparities between rural and urban areas; rural parts of South Africa have 14 times fewer doctors than the national average [7]. 

The period since the mid 1990s has been one of workforce redundancy, vacancy freezes, shortages and cuts in education and provision of training in the public sector [2]. During this time, health outcomes have worsened and inequalities of access to HWs between the public and private sectors, as well as between rural and urban areas have not improved [2]. By way of illustration, the Joint Venture Initiative, a nongovernmental organization (NGO) that recruits doctors to work in under-served rural areas in South Africa, calculated that one-half of the 2400 medical graduates in 2006 and 2007 would leave the country; of the remaining 1200 doctors, 75% would work in the private sector, leaving 300 to work in the public sector; of those 300, possibly 70 or 2.9% of medical graduates each year would work in the public sector in a rural area [8].

There are diverse nonfinancial factors that influence HWs decisions to work in the private sector, urban centre or to migrate abroad. They either push HWs away or pull them towards the private sector, urban area or destination country [4,8,9]. With a focus that does not ignore remuneration, push factors are motivators that drive HWs away from the public sector. Nonfinancial pull factors attract them to the private sector, urban areas or overseas. While economic factors play a significant role in the decisions of HWs as to where they wish to work [5,10], evidence suggests that remuneration is not the principle motivating factor [11-15]. While most of these studies are from outside South Africa, they all allude to the notion that there is much more to motivation and retention than remuneration. Previous research found that the nonfinancial factors that push HWs from the public sector to the private sector, urban area or destination country are: resource-poor health systems, deteriorating work environments, inadequate medicine and equipment, poor human resource planning, political tension and upheaval, gender discrimination, lack of personal security, HIV and AIDS, poor housing, lack of transport and diminishing social systems [16,17]. Of these, this study focuses on resource-poor health systems and deteriorating work environments. In examining the working conditions and perceptions of HWs, this study aims to promote an understanding of the nonfinancial motivating factors for HW employment decisions in South Africa.

Nonfinancial motivating factors in HWs have received a fair amount of empirical attention in recent times [11-13]. A study in Cyprus compared the strength of four work-related motivators for HWs: job attributes, remuneration, co-workers and achievements [11]. They found that achievements, which encompasses job meaningfulness, earned respect and interpersonal relationships, ranked the highest, followed by remuneration, co-workers and job attributes. Other studies have demonstrated similar findings [12-14]. Intrinsic motivating factors, such as job satisfaction, should therefore be viewed as being just as important as extrinsic factors, such as remuneration, in an effort to retain staff and keep them motivated. Meeting the needs of the employee is the cornerstone of job satisfaction and this is of crucial importance for management, as it is strongly correlated with improvements to the quality of service that the organization provides [18].

The aim of this study is to examine and compare the perceptions of HWs in the public, private and NGO sectors of their working conditions and their intentions to stay or leave their existing position. In doing so, this will identify the nonfinancial push and pull factors associated with the trend away from public-sector employment and will highlight areas on which policy-makers need to focus attention given the efforts of government to retain personnel within the public sector.

## Methods

### Study design, setting, population and sample size

The study utilized cross-sectional data collected in 2009 in KwaZulu-Natal, South Africa. The sample of HWs was selected purposively from three public hospitals, two private hospitals and one NGO hospital in the province, and consisted of professional nurses, staff nurses and nursing assistants.

Questionnaires were administered to HWs in urban and peri-urban areas. The researchers used a proportional sampling method in sourcing participants from each employment type (sector), meaning that the number of participants from a given sector was proportionate to the number of HWs working in that sector. The largest number of HWs were selected from the public sector (*n* = 430), followed by the NGO (*n* = 133) and private (*n* = 131) sectors. A total of 694 HWs participated in the study.

### Survey instrument

The survey instrument was based upon the Health Worker Incentives Survey Immpact Toolkit [19]. Questions included: background (demographic) information, cadre type, job and workplace perceptions, assessment of job satisfaction as well as the desire to change their current employer and or location.

### Statistical analysis

Data analysis was conducted using the Statistical Package for the Social Sciences (SPSS Inc., version 18.0, Chicago, IL, USA). The primary unit of analysis in this study was the type of health-care employer (public, private or NGO). Bivariate analysis was used to compare responses from participants in each of the employer types, with both descriptive and inferential statistical methods used. Chi-square tests were conducted to detect levels of significance in demographic and categorical variables (e.g. sex, gender, level of education, employer type), while the one-way analysis of variance (*F* test) was carried out to test for levels of significance in continuous (numerical) variables, such as age. Participants rated various aspects of their working environment on a 5-point Likert-type rating scale (with 1 being low and 5 being high), and the *F* test was used to test for differences in ratings between participants from the three employer types.

### Research ethics

Ethical approval was obtained from the University of KwaZulu-Natal Humanities Research Ethics Committee (Ethical Approval Number: HSS/0703/07). All participants involved in the study were informed about the nature of the study and what their participation would entail. Electronic and hard copies of the data are stored under lock and key at the Health Economics and HIV and AIDS Research Division office, and are only accessible by the researchers involved in the study who are bound by confidentiality agreements.

## Results and discussion

### Demographics

Table 1 shows that 90% (*n* = 620) of the total sample were female – this was consistent across employer types. No significant difference in age was observed between the three employer types, with the mean age being 36.96 years (standard deviation [SD] = 10.38) (*F* = 1.650, *P* = 0.193). The public sector (37%, *n* = 121) had significantly more professional nurses than the private (23%, *n* = 27) and NGO (24%, *n* = 22) sectors (*χ*^2^ = 14.764, *P* < 0.01). This is consistent with the national profile [17]. Significantly more personnel from the private sector (46%, *n* = 60) compared with the public (31%, *n* = 131) and NGO (29%, *n* = 39) sectors indicated that they were married and living with their spouse (*χ*^2^ = 21.960, *P* < 0.05). HWs in the public (51%, *n* = 207) and NGO (48%, *n* = 60) sectors were more likely than HWs in the private sector (21%, *n* = 26) to have served their current employer for five years or more (*χ*^2^ = 152.490, *P* < 0.001). The majority of the sample either resided in a private dwelling that was rented (37%, *n* = 247), or in a privately owned dwelling (32%, *n* = 216) that they were in the process of purchasing. For 75% of the sample, HW income was either the only income in the home or it made up at least 75% of total household income.

**Table 1:** Participant demographics by employer type (sector)

	**Public**	**Private**	**NGO**	**Total**	**Statistical test**	
Sex					*χ*^2^ = 15.451	*P* = 0.116
Male	13 (54)	4 (5)	8 (10)	10 (69)		
Female	87 (374)	96 (125)	92 (121)	90 (620)		
Cadre					*χ*^2^ = 14.764	*P* < 0.01*
Professional nurse	37 (121)	23 (27)	24 (22)	32 (170)		
Staff nurse	33 (108)	48 (58)	39 (36)	38 (202)		
Nursing assistant	30 (98)	29 (35)	37 (34)	31 (167)		
Marital status					*χ*^2^ = 21.960	*P* < 0.05*
Married, living together	31 (131)	46 (60)	29 (39)	34 (230)		
Married, living apart	4 (17)	4 (5)	3 (4)	4 (26)		
Divorced	5 (20)	8 (11)	4 (5)	5 (36)		
Widowed	6 (25)	3 (4)	7 (9)	6 (38)		
Cohabiting, but not married	3 (13)	5 (7)	5 (7)	4 (27)		
Single	51 (212)	34 (44)	52 (69)	48 (325)		
Years of service					*χ*^2^ = 152.490	*P* < 0.001**
<1 year	12 (48)	63 (78)	22 (27)	23 (153)		
1 to 2 years	17 (67)	4 (5)	11 (14)	13 (86)		
2 to 3 years	8 (32)	7 (9)	10 (12)	8 (53)		
3 to 4 years	7 (30)	4 (5)	2 (3)	6 (38)		
4 to 5 years	5 (22)	<1 (1)	6 (7)	5 (30)		
5 years or more	51 (207)	21 (26)	48 (60)	45 (293)		
Accommodation					*χ*^2^ = 13.845	*P* = 0.180
Private, renting	40 (164)	33 (43)	32 (40)	37 (247)		
Private owned, but paying off loan	31 (129)	38 (49)	30 (38)	32 (216)		
Private, owned & paid up	12 (49)	12 (15)	20 (25)	13 (89)		
Private, family/friend provided	15 (60)	17 (22)	17 (22)	16 (104)		
Employer provided	3 (11)	0 (0)	2 (2)	2 (13)		
Age^a^ (years)	37.24 (10.61)	35.32 (8.59)	37.59 (11.08)	36.96 (10.38)	*F* = 1.650	*P* = 0.193

Data presented as percentage (number). ^a^Data presented as mean (standard deviation).

### Working conditions

Participants were asked to rate various aspects of the conditions in which they work on a 5-point rating scale, with 1 being low and 5 being high. When asked to compare the level of their workload with that of colleagues in other sectors, workers in the public sector (mean = 4.55, SD = 0.88) rated their workload significantly higher than workers in the NGO (mean = 4.33, SD = 0.97) and private (mean = 3.81, SD = 1.20) sectors (*F* = 23.869, *P* < 0.001). Personnel from the public (mean = 4.16, SD = 1.03) and NGO (mean = 4.14, SD = 0.92) sectors reported significantly more stress at work compared with workers in the private sector (mean = 3.73, SD = 1.21) (*F* = 8.131, *P* < 0.001). In general, workers who felt more stressed at work were more likely to consider migrating (*F* = 5.251, *P* < 0.01). Also, workers at public facilities (mean = 2.09, SD = 1.25) rated the level of remuneration as significantly lower than workers in the NGO (mean = 2.23, SD = 1.24) and private (mean = 2.61, SD = 1.06) sectors (*F* = 7.868, *P* < 0.001). Moreover, personnel from the public sector (mean = 2.27, SD = 1.19) rated the standard of their working premises as much lower than workers from the NGO (mean = 3.24, SD = 0.98) and private (mean = 3.89, SD = 1.12) sectors (*F* = 106.806, *P* < 0.001). In addition, public HWs (mean = 2.11, SD = 0.94) rated the adequacy of available human resources available as significantly lower than the NGO (mean = 2.32, SD = 1.02) and private (mean = 3.04, SD = 1.00) sectors rated theirs (*F* = 43.474, *P* < 0.001). Finally, public HWs (68%, *n* = 280) were significantly less likely to have received any in-service training since being employed when compared with workers from the private (83%, *n* = 104) and NGO (87%, *n* = 115) sectors (*χ*^2^ = 27.631, *P* < 0.001). A similar, but more acute trend was observed when they were asked whether they received any such training in the past 12 months (Table 2).

**Table 2:** Working conditions by employer type (sector)

	**Public**	**Private**	**NGO**	**Total**	**Statistical test**	
Participants’ rating^a^ of:						
Workload compared to colleagues in other sectors	4.55 (0.88)	3.81 (1.20)	4.33 (0.97)	4.37 (1.00)	*F* = 23.869	*P* < 0.001**
Frequency of the feeling of stress at work	4.16 (1.03)	3.73 (1.21)	4.14 (0.92)	4.08 (1.06)	*F* = 8.131	*P* < 0.001**
Level of remuneration received for work	2.09 (1.25)	2.61 (1.06)	2.23 (1.24)	2.22 (1.23)	*F* = 7.868	*P* < 0.001**
Overall job satisfaction	3.62 (2.55)	3.05 (4.15)	3.53 (1.19)	3.50 (2.74)	*F* = 1.685	*P* = 0.186
Standard of the working premises	2.27 (1.19)	3.89 (1.12)	3.24 (0.98)	2.76 (1.31)	*F* = 106.806	*P* < 0.001**
Level of human resources	2.11 (0.94)	3.04 (1.00)	2.32 (0.96)	2.32 (1.02)	*F* = 43.474	*P* < 0.001**
Staff turnover rate	3.39 (1.25)	3.22 (1.10)	3.53 (1.32)	3.39 (1.24)	*F* = 1.789	*P* = 0.168
Received in-service training since employed^b^					*χ*^2^ = 27.631	*P* < 0.001**
Yes	68 (280)	83 (104)	87 (115)	74 (499)		
No	31 (130)	16 (20)	12 (16)	25 (166)		
Not sure	1 (4)	2 (2)	1 (1)	1 (7)		
Received in-service training in the last 12 months^b^					*χ*^2^ = 83.465	*P* < 0.001**
Yes	40 (163)	73 (91)	76 (100)	53 (354)		
No	58 (238)	23 (29)	22 (29)	44 (296)		
Not sure	2 (9)	3 (4)	2 (2)	2 (15)		

^a^Mean scores based on a rating scale of 1 to 5 (low to high), presented as mean (standard deviation). ^b^Data presented as percentage (number).

HWs in the public sector especially experience poorer working conditions, as indicated by participants’ self-reports on stress, workload, level of remuneration, standard of work premises, level of human resources and frequency of in-service training. Our findings are supported by others, confirming that several salient push and pull factors play a role in HW plans and decisions to migrate [17,20,21].

### Intention to leave their current employment

HWs in the three sectors differed significantly as to whether or not they were considering alternative employment (*χ*^2^ = 22.925, *P* < 0.001). HWs at NGOs (73%, *n* = 91) featured most prominently, followed by workers in the public sector (59%, *n* = 230), while less than one-half (47%, *n* = 56) of workers in the private sector indicated that they were considering moving. Age significantly predicted the desire to leave, with those seeking alternative employment (mean = 35.23, SD = 8.94) being significantly younger than those who were not (mean = 41.93, SD = 11.91) (*F* = 19.544, *P* < 0.001). Nursing cadre (*χ*^2^ = 3.267, *P* = 0.514), sex (*χ*^2^ = 14.725, *P* = 0.142) and marital status (*χ*^2^ = 18.138, *P* = 0.112) did not significantly predict their desire to leave/stay. Several factors play a role in influencing the flow of HWs away from the public sector. As is evident from the results, there are various push factors associated with this sector, and a number of pull factors associated with the private sector (Table 3).

**Table 3:** Participant demographics by their intention to leave their current employment

	**Considering leaving?**					
	**Yes**	**No**	**Don’t know**	**Total**	**Statistical test**	
Employer					*χ*^2^ = 22.925	*P* < 0.001**
Public	59 (230)	21 (81)	20 (76)	100 (387)		
Private	47 (56)	36 (43)	18 (21)	100 (120)		
NGO	73 (91)	16 (20)	11 (14)	100 (125)		
Sex					*χ*^2^ = 14.725	*P* = 0.142
Male	57 (34)	20 (12)	23 (14)	100 (60)		
Female	60 (340)	23 (129)	17 (96)	100 (565)		
Cadre					*χ*^2^ = 3.267	*P* = 0.514
Professional Nurse	63 (98)	18 (28)	19 (30)	100 (156)		
Staff nurse	59 (111)	22 (42)	19 (36)	100 (189)		
Nursing assistant	57 (89)	26 (41)	17 (26)	100 (156)		
Marital status					*χ*^2^ = 18.138	*P* = 0.112
Married, living together	55 (118)	27 (58)	19 (40)	100 (216)		
Married, living apart	76 (19)	8 (2)	16 (4)	100 (25)		
Divorced	58 (18)	32 (10)	10 (3)	100 (31)		
Widowed	39 (12)	36 (11)	26 (8)	100 (31)		
Cohabiting, but not married	62 (18)	12 (3)	19 (5)	100 (26)		
Single	63 (184)	20 (59)	17 (49)	100 (292)		
Age^a^ (years)	35.23 (8.94)	41.93 (11.91)	36.31 (10.76)	36.93 (10.36)	*F* = 19.544	*P* < 0.001**

Data presented as percentage (number). ^a^Data presented as mean (standard deviation).

Although few studies have explicitly set out to determine the variation of push and pull factors across age groups, it is interesting to note that young HWs between the ages of 20 and 29 were more likely to cite deteriorating working conditions as a reason for wanting to leave South Africa, while older respondents (<60 years old) were less likely to identify heavy workloads as a reason for wanting to emigrate [22]. This is also the case in most developing countries where public-sector health facilities in general are characterized by poor infrastructure, management problems, unequal distribution of resources and high numbers of people seeking treatment, accentuated by the high prevalence of HIV and AIDS in most populations on the continent, and in South Africa in particular [17]. Elsewhere it has been argued that while countries in east and southern Africa may not be in a position to provide financial incentives, there are alternatives that have shown positive results in retaining HWs within their respective public health sectors [23]. Lesotho, Mozambique, Malawi and Tanzania have provided housing to their HWs whilst the provision of transport, childcare, food and employee support centres have all elicited positive HW retention outcomes [23].

### Policy developments

Within South Africa, the need to address the problem of both internal and external movement of HWs led to the development and implementation of the Occupational Specific Dispensation for HWs in the public sector to improve their conditions of service and remuneration. Since 2007 when the Occupational Specific Dispensation strategy was developed, the translation of its objectives into practice has evidently not been satisfactory. In 2011 the Department of Health estimated an annual attrition rate of 25%, which excludes an additional 6% for retirement, death and change of profession [2]. This means that every year 25% of the potential workforce (not just new graduates) move from their current position, out of the South African public health sector [2].

The South African Department of Health Strategy 2010/11–2012/13, in priority area five, called for the re-opening of nursing schools and colleges along with the recruitment of HWs from countries with an excess of these professionals [24]. The Strategy went further in placing emphasis on examining the role of community HWs in the public health-care system [24]. The Human Resources for Health (HRH) Strategy for South Africa 2012–2016 further emphasizes the need to strengthen and professionalize the management of human resources and prioritize health workforce needs [2]. To realize this goal, the government set time horizons for the short (1 to 3 years), medium (3 to 5 years) and long (10 to 20 years) term, during which they plan on achieving specific objectives [2]. In expressing part of their strategic direction for the near to distant future, government has committed itself to the scaling up and revitalization of education, training and research, and to strengthening and professionalizing the management of human resources and prioritizing health workforce needs. Based on the findings of this study, as well as other studies [11-13], these goals, if realized, could go some way in curbing the flow of HWs away from the public sector and to other countries. Based on these findings it will serve to attract others into the profession, and perhaps into the public sector. However, often documents propose effective strategies to achieve goals only for it to be discovered later that these goals were not realized due to the failure of authorities and stakeholders to effectively execute or implement these strategies.

To minimize HW attrition and the resultant negative effects, innovative efforts are required to address the causes of HWs’ dissatisfaction and to further identify the nonfinancial factors that influence HWs’ choices, especially given the inability to increase remuneration within a constrained fiscus. The South African Strategic Plan for HIV, tuberculosis and sexually transmitted infections 2012 to 2016 does call for the need to explore ‘innovative financing’ as a mechanism for raising additional resources for the public health care system [25]. Fryatt suggests a number of avenues that South Africa could explore to raise funds and whilst these resources will not solely be allocated to HRH, they could be used to fund efficacious attraction and retention strategies [25]. The government of Malawi responded to their HW crisis by increasing salaries by 50%, improving working conditions, re-enrolment of retired HWs and investment in training for HRH, all of which yielded positive results [26]. Ghana, for their innovative education and training strategies, were recognized with an award from the Health Worker Migration Policy Council [26]. Limited resources, inadequate education and career opportunities along with weak management systems leads to a shortage of HWs [26]. Further research is needed in those countries that have successfully addressed these issues. South Africa, through policy, has set out ways to address the HRH shortage, but implementation is key to ensure there are adequate staffing levels across cadres and in traditionally under-resourced areas.

### Limitations of study

The study is limited by its use of a cross-sectional design that entailed the gathering of data at only one point in time from nurses in selected health facilities in one province of South Africa. The use of proportional sampling does not allow us to generalize these findings to other types of health professionals. Other categories of health professionals, such as pharmacists, physiotherapists, dentists, occupational therapists and psychologists, who are crucial to the delivery of health-care services, were not included. The generalization of these findings to other nurses across the public, private and NGO sectors is further limited by the fact that the nurses participating in this study do not necessary represent nurses across the country working in these sectors.

## Conclusions

The results of this study highlight the importance of implementing a broad range of nonfinancial incentives that are attractive to HWs and encourage them to remain as HWs and importantly to stay in the public sector. While the study identifies several factors that motivate HWs to move out of health, there is a paucity of evidence on the efficacy of various incentive schemes that address the nonfinancial pull and push factors to move within or out of health. Further examination and analysis are needed to better understand the contributing factors to HW motivation and retention, and to better understand the varying degrees to which different incentives influence HW motivation and job satisfaction. This information is critical for effective workforce planning and policy development in the public health sector.

Incentive packages to attract, retain and motivate HWs should be embedded in comprehensive workforce planning and development strategies in South Africa. The research findings from three public hospitals, two private hospitals and one NGO hospital indicate that improved work premises and career advancement and training opportunities are important. Strategies require examination of the underlying factors for HW shortages, analysis of the determinants of HW motivation and retention, and testing of innovative initiatives for maintaining a well-staffed, competent and motivated health workforce, especially in the public sector. Continued research and evaluation will strengthen the knowledge base and assist the development of effective incentive packages for public HWs.

## Abbreviations

HRH: Human Resources for Health; HW: health worker; NGO: nongovernmental organization

## Competing interests

The authors declare that they have no competing interests.

## Authors’ contributions

GG conceived the study and manuscript, lead the acquisition of data and interpretation of data, and was involved in drafting the manuscript. JG designed the study and contributed to the interpretation of the results and revising the manuscript. SB conducted analysis and was involved in drafting the manuscript. All authors read and approved the final manuscript.
